# Diurnal variation in the core interthreshold zone and its relation to cutaneous sensation threshold zone

**DOI:** 10.1186/s40101-017-0141-y

**Published:** 2017-06-21

**Authors:** Naoshi Kakitsuba, Igor B. Mekjavic

**Affiliations:** 1 Department of Environment and Technology, School of Science and Technology, Meijo University, 468-8502 Shiogamaguchi 1-501, Tenpaku-ku, Nagoya, Aichi Prefecture Japan; 2 Jozef Stefan Institute, Ljubljana, Slovenia

**Keywords:** Body temperature regulation, Shivering, Sweating, Cutaneous sensation threshold, Diurnal variation

## Abstract

**Background:**

The core interthreshold zone (CIZ) is defined as the range between temperatures at the onset of shivering and sweating. Its circadian or diurnal variation has not been extensively studied. The present study examined whether the CIZ is subject to a diurnal rhythm. In addition, according to the previous finding that the CIZ was proportionally correlated with peripheral interthreshold zone (PIZ), it was also examined whether cutaneous sensation threshold zone (CSZ), a determinant of the PIZ, is correlated with the CIZ.

**Methods:**

The CIZ and the CSZ were measured in ten Japanese men who underwent three experiments in a single day on the morning, afternoon, and evening in the 2014 experiment (so-called single-day experiment) and six Japanese men underwent the same experiments on the morning of day 1, the afternoon of day 2, and the evening of day 3 in the 2015 experiment (so-called multiple-day experiment). Air temperature was controlled at 20–24 °C. Each subject wore a suit perfused with 25 °C water at a rate of 600 cm^3^/min and exercised on an ergometer at 50% of their maximum work rate for 10–15 min until their rate of sweating increased. They then remained seated without exercising until their oxygen uptake increased. Rectal temperature, skin temperatures at seven sites, the sweating rate at the forehead, and oxygen uptake were continuously monitored throughout experiment. Cutaneous warm and cold sensation thresholds at three sites were measured using 1- and 2-cm^2^ probes.

**Results:**

The results from the single-day experiment demonstrated a small change in the CIZ and core temperature prior to exercise (*T*_c-init_) whereas those from the multiple-day experiment demonstrated continuous increase in the CIZ and *T*_c-init_. The CSZ measured with a 1-cm^2^ probe was inversely proportional to the average skin temperature at three sites prior to measurement (*T*_sk-av_).

**Conclusion:**

The results suggested that the CIZ may be not dependent on time of a day but T_c-init_ per se and may not be associated with the CSZ.

## Background

The core interthreshold zone (CIZ) is defined as the range between core temperature (*T*_c_) at the onset of shivering and that at the onset of sweating. Constant mean skin temperature (*T*_sk_) is required to be independent of thermal responses due to changes in *T*_sk_. The CIZ is recognized as a powerful tool for evaluating the characteristics of body temperature regulation. Taking advantage of the pioneering work by Mekjavic et al. [1], who proposed a methodology for determining the CIZ using a water bath, Kakitsuba et al. [2] devised an alternative method using water-perfused suits, and have conducted a series of studies to investigate the CIZ. In one previous study [3], it was found that the CIZ was twofold wider in the summer than in the winter, and also found that the CIZ was a nearly twofold wider range at a luminance of 36 cd/m^2^ compared with that observed at 18 cd/m^2^, indicating the possible incorporation of a non-visual pathway into the temperature regulation system. In another previous study [4], it was found that the peripheral interthreshold zone (PIZ) was proportionally correlated with the CIZ. This finding suggested that diurnal variation in the CIZ may be correlated with the cutaneous sensation threshold zone (CSZ), a determinant of the PIZ.

The sweating-to-vasoconstriction interthreshold range has been extensively studied. For example, Stephenson et al. [5] demonstrated that the thresholds for sweating and forearm vasodilation were significantly higher at 16 h: 00 and 20 h: 00 than at 24 h: 00 and 04 h: 00, but the zone did not change significantly over the 24-h day. Tayefeh et al. [6] demonstrated that the interthreshold range at 03 h: 00 was twice that observed at the other study times of 8 h: 00, 15 h: 00, and 20 h: 00. These findings suggest that the circadian rhythm may be more complex than just a shift in the central reference temperature.

A circadian or diurnal variation in the CIZ has not been extensively studied. Thus, similar to the suggested circadian variation in the sweating-to-vasoconstriction interthreshold range, the present study examined a diurnal variation in the CIZ. Considering the previous finding suggesting that the CIZ may be correlated with the CSZ, we also examined whether the CSZ displays diurnal variation.

## Methods

### Subjects

The experiment was performed in a climatic chamber at Meijo University, Nagoya, Japan. Ten Japanese men aged 20 to 23 years old participated in the experiments carried out in the summer of single-day, and six Japanese men aged 21 to 23 years old participated in the experiments conducted in the summer of 2015. No subjects took part in both experiments. All subjects were required to sleep before midnight and wake at 07 h: 00 for the 3 days at home prior to the experimental day to reproduce the daily circadian rhythm they would experience during the experiment. Their activities were monitored by wearing an Acti-heart (CamNtech Co., Ltd.) because they were required not to engage in vigorous exercise for a prolonged period of time.

Prior to the experiment, the subjects’ maximum work capacity was estimated during an incremental load exercise on a cycle ergometer. They were asked to pedal at a rate of 60 rpm, and the work rate was increased incrementally by 10 W/min until the subjects were exhausted or could no longer maintain the required cadence. In addition, based on the procedure outlined by Drinkwater [7], anthropometric measurements of skinfold thickness at multiple sites, including the girth, length, and bone breadth of specific body compartments, were taken for each subject. The obtained values were then used to estimate the regional weights of skin, adipose tissue, skeletal muscle, bone, and residual tissues. The adiposity was estimated from the formula proposed by Kakitsuba and Mekjavic [8]. Body surface area (BSA) was calculated using Kurazumi’s formula [9], as it was developed for Japanese morphology. The mean (± SD) height, weight, BSA, and adiposity for group in the single-day experiment were 174.5 ± 4.9 cm, 63.8 ± 7.7 kg, 1.76 ± 0.09 m^2^, and 0.27 ± 0.06, respectively, whereas they were 173.3 ± 5.63 cm, 62.6 ± 11.0 kg, and 1.74 ± 0.13 m^2^ and 0.29 ± 0.04, respectively, for the group in the multiple-day experiment.

**Fig. 1:**
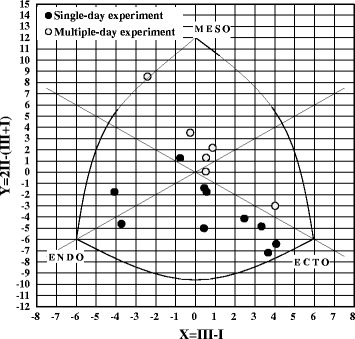
Description of subjects’ somatotypes. The three-component rating of endomorphy (*ENDO*), mesomorphy (*MESO*), and ectomorphy (*ECTO*) for each subject was calculated using the Heath–Carter somatotyping method (1993). Based on the rating of each component, an individual somatotype was determined and is described on the chart. The subjects in the single-day experiment showed a higher ECTO component than those in the multiple-day experiment, whereas the subjects in the 2015 experiment showed a higher MESO component than those in the single-day experiment

A three-component rating of endomorphy (ENDO), mesomorphy (MESO), and ectomorphy (ECTO) for each subject was calculated using the Heath–Carter somatotyping method [10]. Based on the rating of each component, an individual somatotype was determined and is described in Fig. 1.

In the 2014 experiment, so-called single-day experiment, the subjects underwent three consecutive experiments in a single day: morning (08 h: 30–11 h: 00), afternoon (13 h: 00–15 h: 30), and evening (17 h: 00–19 h: 30). Following the morning and afternoon tests, the subject was passively rewarmed for 1 h with a heater to restore core temperature. In the 2015 experiment, so-called multiple-day experiment, the subjects underwent the experiment on the morning of day 1, the afternoon of day 2, and the evening of day 3 without any rewarming procedure.

All subjects gave their informed consent to participate in the study and were fully aware that they could withdraw from the study at any time without prejudice. The study protocol was approved by the ethics review committee at Meijo University.

### Experimental protocol

Because the lighting and season effect to the CIZ was confirmed in the previous study [3], illuminance was carefully controlled to be at 1000 lx, and all experiments were conducted from August to September as described in the experimental protocol of our previous study [4].

**Fig. 2:**
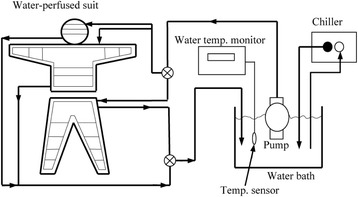
A subject wearing a water-perfused suit. This photograph is of a subject wearing a water-perfused suit and exercising at 50% of his maximum work rate on a cycle ergometer

The *T*_sk_, as calculated using the equation proposed by Hardy and DuBois [11], was maintained at 28 °C using the water-perfused suit shown in Fig. 2. Subjects wearing a water-perfused suit and stayed on a cycle ergometer for 5 to 10 min without exercise until *T*_sk_ decreased to 28–29 °C. They then commenced exercising at 50% of their maximum work rate on the cycle ergometer. The exercise was terminated at the onset of sweating, which occurred after 10–15 min of exercise. The subjects then remained seated on the cycle ergometer for an additional 100 min. The onset of shivering was observed when the oxygen uptake started to increase during the last part of the test, while the *T*_sk_ remained at 28 °C. The CIZ was defined as the rectal temperatures (*T*_re_) at which the sweating rate (S_wr_) and oxygen uptake were elevated above their median resting levels.

### Measurements

The *T*_re_ and skin temperatures at seven sites (forehead, forearm, back of hand, abdomen, chest, anterior thigh, and calf) were monitored with thermistors, and the values were stored every 10 s using a data logger system (Cadac2 Model 9200A; Cadac, Tokyo, Japan). In the case of rectal temperature, a thermistor was inserted 15 cm at the anal verge. The S_wr_ was measured at the forehead with a sweat rate monitor (Model SKD-4000; Skinos Co., Ltd., Nagoya, Japan). Oxygen uptake was monitored with a gas analyzer (Respiromonitor RM-300i; Minato Medical Science Co., Ltd., Tokyo, Japan).

**Fig. 3:**
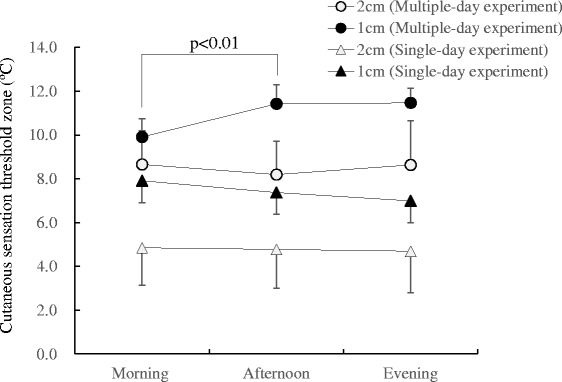
Diagram of the cooling system for the water-perfused suit. A chiller cooled the water in the bath and a pump supplied cool water to the vinyl tubes incorporated in the suit. After the water was perfused through the tubes, it was returned to the water bath and cooled again

The maintenance of *T*_sk_ while simultaneously extracting 120 W/m^2^ of heat was achieved by having the subjects wear a water-perfused suit (Cool Tubesuit; Med-Eng Systems, Inc., Ottawa, Ontario, Canada). The water perfusing the suit was pumped at a rate of 600 cm^3^/min (Water Pump Model Super Tepcon; Terada, Tokyo, Japan) from a bath in which the water temperature was maintained at 25 °C by a heat exchanger (Cool Mate Model TE-105M; Toyo Seisakusho Co., Tokyo, Japan). A diagram of the cooling system is shown in Fig. 3.

Before each test, cutaneous warm and cool sensation thresholds were measured at the anterior forearm, posterior forearm, and anterior thigh by using a thermal stimulator that was controlled by a Peltier element (Intercross-230, Intercross, Co.). In order to prove no or small difference of diurnal variation in peripheral thermosensitivity regardless of the size of the thermode, two probes were used so that the contact area when applied to the skin was either 10 × 10 mm or 20 × 20 mm. Once the probe surface temperature became equal to the skin temperature, i.e., adaption temperature, it was changed at a rate of 0.1 °C/s and continuously changed until the subject felt a warm or cool sensation. The cutaneous sensation threshold zone (CSZ) was then determined as the temperature range between the warm and cool sensation thresholds.

### Statistical analysis

All physiological variables measured are presented as means ± SD. A comparison of the variables between the three daytime periods was analyzed using repeated measures ANOVA. The Bonferroni test was used for post-hoc analysis of significant differences set at *p* < 0.05.

## Results

### Diurnal variation in the CSZ and the average skin temperature prior to measurement

**Fig. 4:**
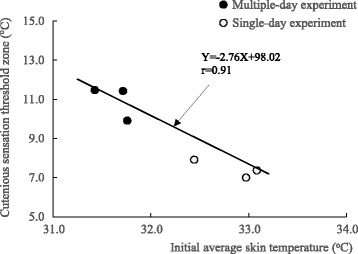
Diurnal variation in the cutaneous sensation threshold zone measured with 1- and 2-cm^2^ probes. The CSZ measured with the 2-cm^2^ probe showed no diurnal variation. By contrast, the CSZ measured with the 1-cm^2^ probe showed a significant increase (*p* < 0.01) from the morning to the afternoon sessions in the multiple-day experiment

**Fig. 5:**
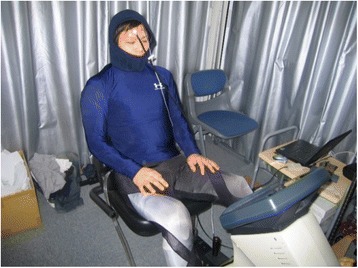
Relationship of the cutaneous sensation threshold zone measured with the 1-cm^2^ probe to the average skin temperature prior to measurement. The CSZ is inversely correlated with the average skin temperature prior to measurement. Thus, a higher skin temperature is associated with a narrower CSZ

The CSZ and average skin temperature at three sites prior to measurement (*T*_sk-av_) in the two experiments are indicated in Fig. 4. The CSZ as measured with a 2-cm^2^ probe showed no diurnal variation. By contrast, the CSZ measured with the 1-cm^2^ probe showed a continuous decrease in the single-day experiment and a significant increase (*p* < 0.01) from morning to afternoon in the multiple-day experiment. Because the CSZ measured with the 1-cm^2^ probe showed diurnal variations that were due mainly to a change in the warm sensation threshold, the CSZ values were compared with the *T*_sk-av_. As indicated in Fig. 5, the CSZ was inversely correlated with the *T*_sk-av_.

### Diurnal variation in the CIZ, sweating and shivering thresholds, and rectal temperature before exercise

The results for the diurnal variation in the CIZ, sweating and shivering thresholds, and rectal temperature before exercise in the two experiments are shown in Tables 1 and 2. The results from the single-day experiment revealed that the shivering *T*_c_ threshold increased from morning to afternoon and remained unchanged from afternoon to evening, whereas the sweating *T*_c_ threshold was almost unchanged. As a result, the CIZ decreased during the day, although the differences were not statistically significant.

**Table 1:** Results from the single-day experiment

Subjects	Morning				Afternoon				Evening			
	CIZ (°C)	*T*_c-sweating_ (°C)	*T*_c-shivering_ (°C)	*T*_c-int_ (°C)	CIZ (°C)	*T*_c-sweating_ (°C)	*T*_c-shivering_ (°C)	*T*_c-int_ (°C)	CIZ (°C)	*T*_c-sweating_ (°C)	*T*_c-shivering_ (°C)	*T*_c-int_ (°C)
a	0.50	36.86	36.36	37.08	0.48	36.86	36.38	36.96	0.46	36.78	36.32	36.62
b	0.27	37.05	36.78	37.18	0.59	37.12	36.53	37.02	0.12	36.85	36.73	36.59
c	0.03	36.78	36.75	36.96	0	36.94	36.96	37.02	0	37.04	37.08	37.14
d	0.23	36.83	36.6	36.55	0	37.05	37.05	36.57	0.05	37.04	36.99	37
e	0.3	36.88	36.58	36.71	0.47	37.01	36.54	36.82	0.54	36.93	36.39	36.56
f	0.05	37.01	36.96	37.05	0	37.16	37.17	36.6	0.07	37.17	37.1	37.1
g	0.5	36.27	35.77	36.69	0	37.03	37.05	37.01	0.15	36.94	36.79	36.64
h	0.26	36.97	35.71	37.00	0.21	37.11	36.90	36.93	0.21	37.00	36.89	36.84
i	0.17	37.8	37.63	37.58	0.07	37.25	37.18	37.15	0.38	37.46	37.08	37.2
j	0.38	37.38	37	37.34	0.2	37.52	37.32	37.28	0.04	37.41	37.37	37.2
Means ± SD	0.27 ± 0.17	36.98 ± 0.42	36.71 ± 0.50	37.02 ± 0.33	0.20 ± 0.25	37.10 ± 0.19	36.91 ± 0.34	36.94 ± 0.24	0.20 ± 0.20	37.07 ± 0.31	36.87 ± 0.34	36.89 ± 0.28

*CIZ* core interthreshold zone, *T*_*c-sweating*_ sweating threshold, *T*_*c-shivering*_ shivering threshold, *T*_*c-int*_ core temperature before exercise

**Table 2:** Results from the multiple-day experiment

Subjects	Morning				Afternoon				Evening			
	CIZ (°C)	*T*_c-sweating_ (°C)	*T*_c-shivering_ (°C)	*T*_c-int_ (°C)	CIZ (°C)	*T*_c-sweating_ (°C)	*T*_c-shivering_ (°C)	*T*_c-int_ (°C)	CIZ (°C)	*T*_c-sweating_ (°C)	*T*_c-shivering_ (°C)	*T*_c-int_ (°C)
A	0.22	37.32	37.10	37.25	0.04	37.22	37.18	37.22	0.06	36.99	36.93	37.30
B	0.04	36.80	36.76	36.75	0.33	37.20	36.87	37.10	0.61	37.63	37.02	37.34
C	0.03	36.79	36.76	36.70	0.59	37.28	36.69	37.00	0.35	37.20	36.85	37.36
D	0.06	36.75	36.69	37.01	0.00	36.50	36.50	36.50	0.01	36.84	36.83	36.86
E	0.40	37.18	36.78	37.05	0.09	37.48	37.39	37.50	0.71	37.76	37.05	37.60
F	0.05	37.26	37.21	37.13	0.19	37.32	37.13	37.30	0.24	37.68	37.44	37.50
Means ± SD	0.13 ± 0.15	37.02 ± 0.26	36.88 ± 0.22	36.98 ± 0.22	0.21 ± 0.22	37.17 ± 0.34	36.96 ± 0.33	37.1 ± 0.34	0.33 ± 029	37.35 ± 0.39	37.07 ± 0.21	37.33 ± 0.25

*CIZ* core interthreshold zone, *T*_*c-sweating*_ sweating threshold, *T*_*c-shivering*_ shivering threshold, *T*_*c-int*_ core temperature before exercise

The results from the multiple-day experiment revealed that the shivering and sweating *T*_c_ thresholds increased continuously from morning to evening. The results also showed that the CIZ increased throughout the day, although these increases were not statistically significant because of large individual differences. The results also indicated that the CIZ values obtained from the multiple-day experiment showed no significant differences with significantly higher *T*_c-init_ (*p* < 0.01) than those obtained from the single-day experiment.

## Discussion

To determine whether there is a diurnal variation in the CIZ, the *T*_c_ was maintained to be consistent as possible throughout the day by conducting the experiments sequentially on the same day in the single-day experiment. As indicated in Table 1, the CIZ showed almost no diurnal variation in association with almost no diurnal variation of *T*_c-init_. The typical *T*_c_ diurnal rhythm test was then repeated by conducting the experiments on different days in the multiple-day experiment. As indicated in Table 2, the CIZ increased from morning to evening in association with diurnal variation of *T*_c-init_. Thus, the CIZ appeared to be dependent on the *T*_c-int_ per se.

Kakitsuba et al. [4] reported a strong correlation between the CIZ and the temperature difference between *T*_c-init_ and the core shivering threshold. In other words, the CIZ mainly depends on a decrease in *T*_c_ from *T*_c-init_. In the single-day experiment, however, the results suggested that the CIZ depended on an increase in *T*_c_ from *T*_c-init_, with the exception of the morning period. This means that sweating did not occur until *T*_c_ increased 0.2 °C from *T*_c-init_ when *T*_c_ decreased throughout the day, specifically during the afternoon and evening. In the multiple-day experiment, each subject underwent once per day, using a randomized schedule to measure physiological responses associated with the standard *T*_c_ diurnal rhythm. As indicated in Table 2, the CIZ depended primarily on a decrease in *T*_c_ from *T*_c-init_ without exception. This means that shivering did not occur until *T*_c_ decreased 0.2 °C from *T*_c-init_ when *T*_c_ increased throughout the time periods examined. Thus, the CIZ responded differently to the diurnal variation in *T*_c_, and the CIZ appeared to be directly correlated with *T*_c-init_, despite the two groups showing a difference in *T*_c-init_ because of the different experimental protocols.

The morning *T*_c-init_ in the multiple-day experiment was significantly higher than that in the single-day experiment (*p* < 0.01). As described in the Methods section, the groups in the single-day and multiple-day experiments showed almost no difference in physical characteristics, with the exception of somatotype components, which showed a clear difference, as indicated in Fig. 1. The subjects in the multiple-day experiment exhibited a higher MESO component than those in the single-day experiment, who exhibited a higher ECTO component. This may have contributed to the difference in the morning *T*_c-init_ because of the relatively higher muscularity of the subjects in the multiple-day experiment compared with those in the single-day experiment.

Tayefeh et al. [6] reported that the sweating-to-vasoconstriction interthreshold range at 03 h: 00 (early morning) was twice that observed at the other study times (*p* < 0.05), leading them to conclude that diurnal variation alters the thermoregulatory target temperature and plays a role in the precision of body temperature control. The results from the multiple-day experiment are consistent with those of Tayefeh et al. [6], who controlled *T*_c_ to reproduce a standard circadian rhythm, which exhibits continuously increasing *T*_c_ from morning to evening. However, the results from the single-day experiment showed almost no diurnal variation in *T*_re_, with almost no diurnal variation in *T*_c-int_. For this reason, the CIZ is not dependent on the diurnal *T*_c_ rhythm but on *T*_c_ per se.

The results of our previous study [2] showed that the CIZ is proportionally correlated with the peripheral interthreshold zone (PIZ). Since the CSZ is thought to be a determinant factor of the PIZ, diurnal variation in the CIZ may be expected to be proportionally correlated with the CSZ. In both experiments, the CSZ measured with a 2-cm^2^ probe showed almost no diurnal variation, whereas the CSZ measured with a 1-cm^2^ probe showed a significant change, as indicated in Fig. 5. According to a previous report [12], the CSZ is inversely proportional to the contact area. Because the difference is due only to its sensitivity, the change in the CSZ can be detected no matter what size probe is used. Recent studies examining the CSZ used a 2.5-cm^2^ probe to demonstrate race [13] and age-related [14, 15] differences. Jakovljevic and Mekjavic [16] used a 12.5-cm^2^ probe to determine cutaneous temperature sensitivity, and Golja et al. [17] used a 24-cm^2^ probe to show gender differences. Although probes with different contact areas were used in these previous studies, cutaneous temperature sensitivity could be compared under various thermal conditions and subject groups as long as it was measured at a constant rate change of 1 °C/s. According to these findings, therefore, different responses to a change in the measured skin temperature by using probes of different sizes were not anticipated. Nevertheless, higher skin temperature appears to be associated with a narrower CSZ only when the cutaneous sensation threshold was measured with the 1-cm^2^ probe. Strigo et al. [18] examined whether human pain perception may be dependent on ambient temperature using a 1-cm^2^ contact thermode. Ambient temperature was controlled at 15, 25, and 35 °C. The results indicated that a decrease in skin temperature following exposure to cool environments reduces thermal pain, and thus may support the result from the present study as indicated in Fig. 5.

The standard *T*_c_ diurnal rhythm is a progressive increase in *T*_c_ in the morning, followed by a more gradual increase during the afternoon and then a relatively constant *T*_c_ during the evening. However, this *T*_c_ diurnal rhythm may not always exist because, as reported by Kakitsuba and White [19], both *T*_c_ and *T*_sk_ are easily affected by change in ambient temperature during the afternoon and evening. When the human body is exposed to cool or cold conditions, the CIZ becomes narrower due to lower *T*_c_ but the CSZ is expected to be wider with lower *T*_sk_, if a change in the CSZ measured with a 1-cm^2^ probe can be accepted as reliable. This implies that the afferent thermoregulatory pathway, as reflecting thermal perception, may not promptly mediate a metabolic response. On the other hand, when the human body is exposed to warm or hot conditions, the CIZ becomes wither with higher *T*_c_ but the CSZ is expected to be narrower with higher *T*_sk_. This implies that the afferent thermoregulatory pathway may promptly mediate a sweating response. Thus, the results from the present study suggest that the cutaneous contributions to control of sweating and shivering may change with thermal conditions.

## Conclusion

The results showed that the CIZ appeared to be dependent on core temperature prior to exercise (*T*_c-init_) not on the time of day. Therefore, diurnal variation in the CIZ was not proved. In addition, the CSZ as measured with a 1-cm^2^ probe was inversely proportional to the average skin temperature at three sites prior to measurement (*T*_sk-av_). These results suggested that the CIZ subject to *T*_c_ may not be associated with the CSZ subject to the *T*_sk-av_.

## Abbreviations

BSA: Body surface area
CIZ: Core interthreshold zone
CSZ: Cutaneous sensation threshold zone
ECTO: Ectomorphy
ENDO: Endomorphy
*E*_sk_: Sweating rate
MESO: Mesomorphy
PIZ: Peripheral interthreshold zone
*T*_sk_: Mean skin temperature
*T*_c_: Core temperature
*T*_c-init_: Core temperature prior to exercise
*T*_re_: Rectal temperature
*T*_sk-av_: Average skin temperature at three sites prior to measurement
